# Human Papillomavirus Up-Regulates MMP-2 and MMP-9 Expression and Activity by Inducing Interleukin-8 in Lung Adenocarcinomas

**DOI:** 10.1371/journal.pone.0054423

**Published:** 2013-01-21

**Authors:** Ming-Yuh Shiau, Li-Ching Fan, Shun-Chun Yang, Chang-Hui Tsao, Huei Lee, Ya-Wen Cheng, Li-Chuan Lai, Yih-Hsin Chang

**Affiliations:** 1 Hungkuang University, Taichung, Taiwan, Republic of China; 2 Institute of Medical & Molecular Toxicology, Chung Shan Medical University, Taichung, Taiwan, Republic of China; 3 School of Medical Laboratory and Biotechnology, Chung Shan Medical University, Taichung, Taiwan, Republic of China; 4 Graduate Institute of Life Sciences, National Defense Medical Center, Taipei, Taiwan, Republic of China; 5 Graduate Institute of Cancer Biology and Drug Discovery, Taipei Medical University, Taipei, Taiwan, Republic of China; 6 Department of Biotechnology and Laboratory Science in Medicine, National Yang-Ming University, Taipei, Taiwan, Republic of China; Kagoshima University Graduate School of Medical and Dental Sciences, Japan

## Abstract

Human papillomavirus (HPV) infection is associated with non-smoking female lung cancer. Our previous report demonstrated that HPV 16 promotes lung tumor cell progression by up-regulating interleukin-17 (IL-17). IL-17 and its downstream signaling mediator, interleukin-8 (IL-8), have been implicated to modulate a variety of pro-angiogenic factors and play important roles in tumor angiogenesis and metastasis. Accordingly, we hypothesized that HPV infection may potentiate tumorigenic and metastatic characteristics of the infected cells through IL-8. The goal of the present study was to determine whether HPV infection in lung adenocarcinoma cells can promote the expression of IL-8 and metalloproteinases (MMPs) to make the transformed cells equipped with angiogenic and metastatic characteristics. The expression of IL-8 and MMPs in HPV 16 E6-transfected H1299 cells was analyzed to examine the hypothesis. HPV 16 E6 up-regulates pro-angiogenic MMP-2 and MMP-9 through inducing IL-8 expression in lung cancer cells. The results indicate that, in addition to cell proliferation-related machinery, HPV infection promotes the expression and activities of angiogenic and metastatic molecules in lung adenocarcinoma cells. The cytokines induced by HPV infection may work together to confer the malignant and tumorigenic potentials on the infected cells by promoting machineries of growth, angiogenic and metastatic characteristics.

## Introduction

Lung cancer is one of the most common causes of cancer death in developed countries. The tumorigenesis and disease progression of lung cancer are a complicated process including cell transformation, evasion of host defenses, angiogenesis, invasion and metastasis. Angiogenesis is not only regulated by a complex interaction among various growth factors and cytokines, but also influenced by proteolytic enzymes such as matrix metalloproteases (MMPs) and distribution of extracellular matrixes [1]. Recent evidence suggests that angiogenesis is related to poor prognosis in lung cancer [2]–[5].

Our previous studies document that human papillomavirus (HPV) infection is associated with non-smoking Taiwanese female lung cancer [6], [7]. The E6 antigens of HPV 16 up-regulate interleukin-6 (IL-6) and anti-apoptotic Mcl-1 which, subsequently, may promote the tumor progression of HPV-infected lung cancer [8]. More recently, we further reveal that CD4+ T cell derived pro-inflammatory cytokine interleukin-17 (IL-17) is elevated in HPV-infected lung adenocarcinoma cells [9]. Evidences show that tumor cell-derived IL-17 may promote *in vivo* tumor growth and potentiate angiogenesis [10]. The CD4+ T cell derived cytokine IL-17 promotes tumor angiogenesis via not only stimulating vascular endothelial cell migration but also inducing a variety of pro-angiogenic factors, particularly IL-8, that lead to the imbalance between angiogenesis activators and inhibitors within vascular microenvironment [10].

IL-8 is a potent angiogenic factor which is associated with metastasis in several cancers [11]–[14]. Study from Bequet-Romero et al. reported that conditioned media of HPV-positive cells are able to induce pro-angiogenic IL-8 expression that supports tumor growth and invasion in human umbilical vein endothelial cells [15]. They also demonstrated that IL-8 is predominantly detected in lung tumor cells [15] and the elevated IL-8 is correlated with angiogenesis, tumor progression and poor survival in non-small cell lung cancer (NSCLC) [16]–[18]. Tumor-derived IL-8 levels are correlated with the growth rate of human NSCLC cells in SCID mice [19]. *In vitro* and animal studies [19], [20] show that lung cancer cell proliferation and growth can be inhibited by antagonizing IL-8 effects. Accumulating evidence demonstrates that serum IL-8 levels are not only elevated in patients with advanced NSCLC, but also associated with patients’ clinicopathologic characteristics, angiogenesis, tumor progression and prognosis [16]–[21]. The above findings suggest that biological effects of IL-8 during lung tumorigenesis are mainly promoting angiogenesis. Inone et al. reported that by overexpressing IL-8 in human prostate cancer cells, the concomitantly up-regulated MMP-9 expression and collagenase activities would promote tumor cell growth and metastasis [22]. Furthermore, endothelial cell angiogenesis is promoted under IL-8 treatment through boosting MMP-2 and MMP-9 expression [23]. These results further indicate that regulation of angiogenesis by IL-8 in tumor cells is, at least in part, through induction of MMP-2 and MMP-9 expression.

According to the bioactivity of IL-17 to promote tumorigenesis and the expression of down-stream cytokines as well as the significant correlation between IL-17 expression and HPV infection in lung tumors, we hypothesized that HPV-induced IL-17 may potentiate tumorigenic and metastatic characteristics of the infected cells through its downstream signaling mediator IL-8. Therefore, the present study examined the expression and activity of metastasis-related proteins in HPV-transfected H1299 lung adenocarcinoma cells to verify the above hypothesis. In this regard, pro-angiogenic IL-8, MMP-2, and MMP-9 expressions were examined in HPV 16 E6- transfected H1299 cells. Our results reveal that HPV 16 E6 in H1299 cells can significantly up-regulate pro-angiogenic MMP-2 and MMP-9 through inducing IL-8.

## Materials and Methods

### Cell Culture and HPV 16 E6 Transfection

Human lung adenocarcinoma H1299 cells were obtained from American Type Culture Collection (Manassas, VA, USA) and cultured in RPMI-1640 supplemented with 10% FBS, 2 mM glutamine, 100 units/mL penicillin, and 100 mg/mL streptomycin (Life Technologies, Inc.) at 37°C and 5% CO_2_ [9]. Full length E6 of HPV 16 was amplified by PCR from HPV 16 genome-containing CasKi cells as previously described [8]. The resulted PCR products were purified with GENECLEAN III kit (Qbiogene, Irvine, CA, USA) and cloned into a eukaryotic expression vector pIND/V5-His-TOPO (Invitorogen, California, USA). DNA prepared from the resultant recombinants were transfected into H1299. On the day prior to transfection, cells were grown to 80% confluency in RPMI-1640 medium, and the cells were cotrasfected with either pIND/TOPO/lacZ and pVgRXR_-_verB (H1299-pIND) or pIND/TOPO-HPV 16 E6 and pVgRXR_-_verB (H1299-HPV16E6) in 40 *u*L DOTAP Liposomal (Roche Diagnostics GmbH, Mannheim, Germany) and 100 *u*L HBS buffer (20 mM HEPES, 150 mM NaCl, pH 7.4). The culture media was aspirated, and fresh RPMI-1640 medium was added after 6 hours of incubation, then the transfects were maintained in medium containing 500 *u*g/mL G418.

### RNA Extraction and RT-PCR

Total RNA was isolated using TRIZOL reagent (Life Technologies, Gaithersburg, MD). Briefly, cDNA was synthesized using total RNA (3 *u*g), oligo dT primer (200 *p*mol), and 5× MMLV RT. Then the PCR conditions for each target gene were optimized before analysis, with the respective number of PCR amplification cycle, annealing temperature and primer sets listed in Table 1. All RT-PCR reactions of target genes were carried out with BIO-RAD PCR iCYCLER. Amplified products were identified by electrophoresis and then stained with ethidium bromide.

**Table 1:** PCR conditions for each target gene used in this study.

Gene	Sequence (5′-3′)	Product size (bp)	Annealing temperature	Number of PCR cycle
*HPV16 E6*	ACT GCA ATG TTT CAG GAC CC TCA GGA CAC AGT GGC TTT TG	344	54.9	28
*IL-8*	TTC TGC AGC TCT GTG TGA AGG TAT GAA TTC TCA GCC CTC TTC	248	59	28
*MMP-2*	GGC CCT GTC ACT CCT GAG AT GGC ATC CAG GTT ATC GGG GA	500	62	32
*MMP-9*	CAA CAT CAC CTA TTG GAT CC CGG GTG TAG AGT CTC TCG CT	600	62	32
*GAPDH*	ACC ACA GTC CAT GCC ATC AC TCC ACC ACC CTG TTG CTG TA	452	58	28

### Western Blot Analysis

Cell lysates were harvested using protease inhibitor-containing lysis buffer (50 mM Tris, 0.1% Triton X-100, 107 mM NaCl, 2 mM EDTA, 1 mM Na_3_VO_4_, 1 mM phenylmethylsulfonyl fluoride, 10 *u*g/*u*L apotinin and 10 *u*g/mL leupeptin, pH 7.5) and quantitated by using Bio-Rad Bradford protein assay kit. Equal amounts of proteins (30 *u*g/lane) were separated on 12% SDS-PAGE and electrotransfered to PVDF membranes. The membranes were blocked with 1× Tris buffered saline-Tween buffer (TTBS; 10 mM Tris-HCl, pH 7.5, 150 mM NaCl, 0.05% Tween-20) containing 5% skim milk and then probed with primary antibody for HPV-C1P5 (Santa Cruz, CA, USA), IL-8 (Santa Cruz, CA, USA), and *ß*-actin (Sigma), respectively. The membrane was then washed 3 times and incubated with secondary antibody conjugated with HRP for 90 min at room temperature. After washing 5 times with TTBS, the membrane was developed with enhanced chemiluminescence kit (ECL kit, Amersham Biosciences, Piscataway, NJ, USA). *ß*-actin in all of the samples was probed to exclude false-negative results.

### Zymography

Culture media were harvested after parental H1299, H1299-pIND and H1299-HPV16E6 cells were cultured in serum-free RPMI 1640 with or without 5 *u*M ponasterone A (PonA) and 100% alcohol (Alc), respectively, for 24 hours. The activities of MMP-2 and MMP-9 in the collected media were measured as previously described [24]. Briefly, samples were prepared with standard SDS-gel-loading buffer containing 0.01% SDS without *ß*-mercaptoethanol and heating. The prepared samples were subjected to electrophoresis with 8% SDS-PAGE containing 0.1% gelatin. Following electrophoresis, the gels were washed in 2.5% Triton X-100 at room temperature to remove SDS, incubated in 100 mL reaction buffer (40 mM Tris-HCl, pH 8.0, 10 mM CaCl_2_, 0.02% NaN_3_) for 24 h at 37°C and stained with Coomassie brilliant blue R-250 containing 50% methanol and 10% acetic acid. Gelatinolytic activities were visualized by negative staining with 20% methanol and 10% acetic acid. Clear bands indicated gelatinolytic activities were present at the expected molecular weight for MMP-2 and MMP-9.

### Enzyme-linked Immunosorbent Assay

Culture media were harvested after parental H1299, H1299-pIND and H1299-HPV16E6 cells were cultured in serum-free RPMI 1640 with or without 5* u*M PonA and 100% Alc for 24 hours, respectively. Cell-free supernatants were collected and stored at −20°C until analysis. Levels of IL-8, MMP-2 and MMP-9 were measured by ELISA kits (R&D Systems).

### Statistical Analysis

The significance of the data was determined by χ^2^-test or Fisher’s exact test. A value of p<0.05 was considered as significant.

## Results

### IL-8 Expression is Up-regulated in HPV 16 E6-transfected H1299 Cells

**Figure 1:**
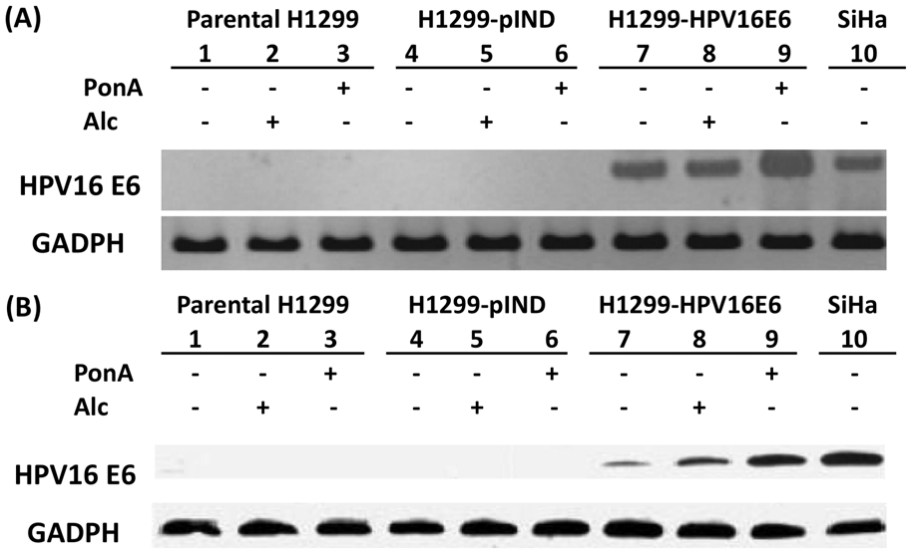
Expression of HPV 16 E6 in H1299 lung cancer cells. HPV 16 E6 expression levels of H1299-HPV16E6 induced by 5 µM ponasterone A (PonA) was significantly higher than that of parental H1299, H1299-pIND and H1299-HPV16E6 treated with 100% alcohol (Alc) or PonA after 24 hours. (A) RNA was isolated and subjected to RT-PCR analysis using specific primers to HPV 16 E6 and GAPDH. (B) Total cell extracts were used to detect HPV 16 E6 and *β*-actin by Western blot analysis. HPV 16 E6-containing SiHa cervical cancer cells were used as positive control (lane 10).

The study started with establishing HPV-transfected H1299 lung adenocarcinoma cells for the purpose of investigating the effects of HPV infection on IL-8 expression. HPV 16 E6 RNA and protein levels were analyzed in parental H1299 cells, control vector-transfected H1299 cells (H1299-pIND) and full-length HPV 16 E6-transfected H1299 cells (H1299-HPV16E6). As shown in Figure 1, HPV 16 E6 expression could not be detected in parental H1299 and H1299-pIND cells. On the contrary, both the expression of HPV 16 E6 RNA (Fig. 1A) and proteins (Fig. 1B) were highly induced upon the treatment of inducing agent PonA, compared with the Alc-treated counterparts. Therefore, HPV 16 E6 expression could be successfully controlled by using this induction system.

**Figure 2:**
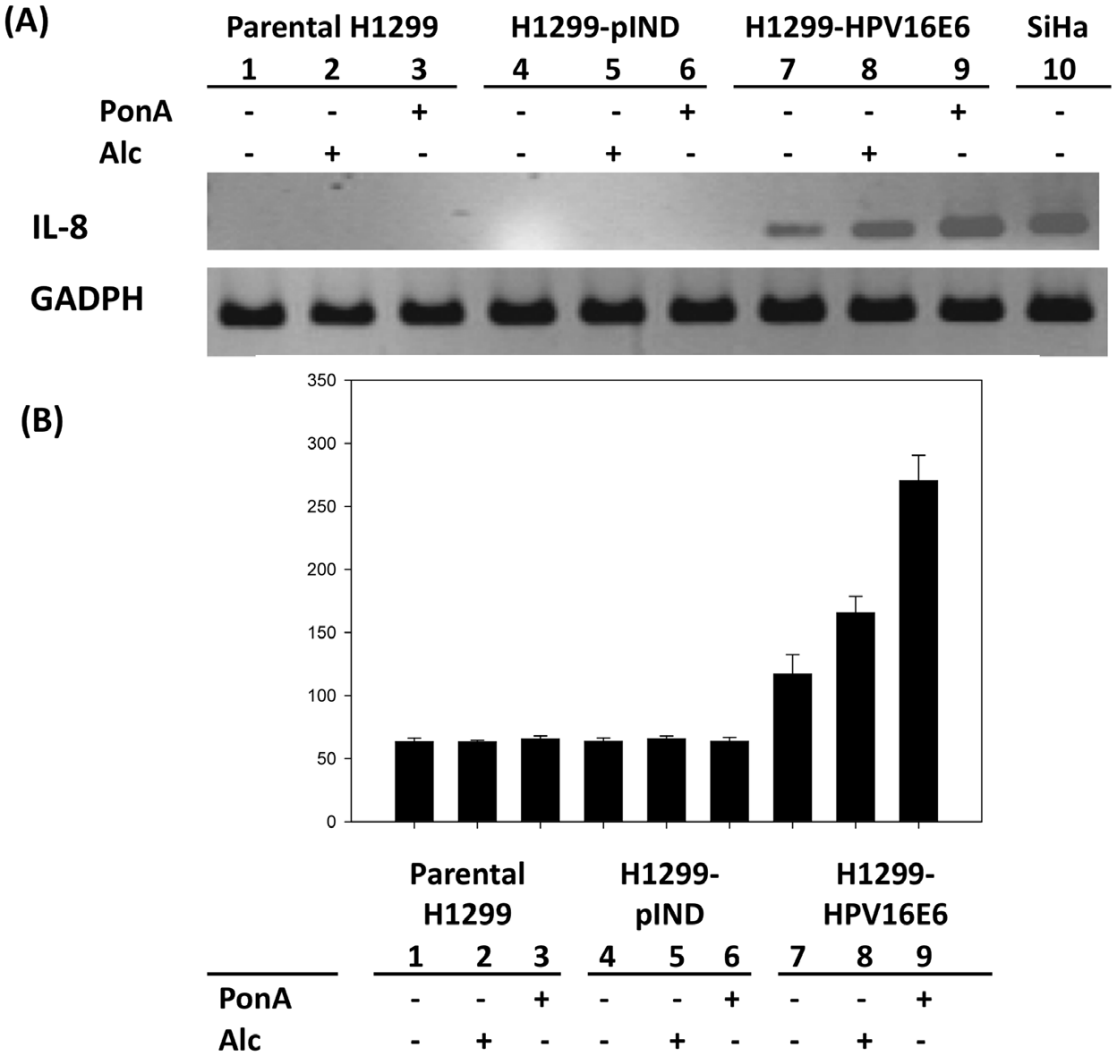
HPV 16 E6 induces IL-8 production in H1299 lung cancer cells. IL-8 expression levels of H1299-HPV16E6 induced by PonA were significantly higher than that of parental H1299, H1299-pIND and H1299-HPV16E6 treated with Alc or PonA after 24 hours. (A) RNA was isolated and subjected to RT-PCR analysis using specific primers to IL-8. HPV 16 E6-containing SiHa cervical cancer cells were used as positive control (lane 10). (B) IL-8 levels in cell culture supernatants were analyzed by ELISA (*P<0.05 by *t*-test).

The putative effects of HPV infection on IL-8 expression were subsequently analyzed. As shown in Figure 2A, IL-8 RNA expression was significantly up-regulated in H1299-HPV16E6 cells upon PonA treatment. IL-8 secretion of the parental H1299 cells and H1299-pIND, either in the presence of PonA or Alc treatment, was approximately 65 pg/mL (Fig. 2B and Table S1). Notably, the IL-8 levels of H1299-HPV16E6 were dramatically elevated about 2.5 folds in E6 transfectants by PonA stimulation (270.59±19.88 pg/mL; Figure 2B and Table S1). Whereas, the IL-8 levels of the E6 transfectant without inducing agent and under Alc treatment was 117.10±15.24 pg/mL and 165.53±13.10 pg/mL, respectively (Fig. 2B and Table S1). These results indicated that IL-8 is significantly up-regulated accompanying with HPV 16 E6 expression in lung cancer cells.

### MMP-2 and MMP-9 Expression and Activities are Up-regulated in HPV16 E6-transfected H1299 Cells

**Figure 3:**
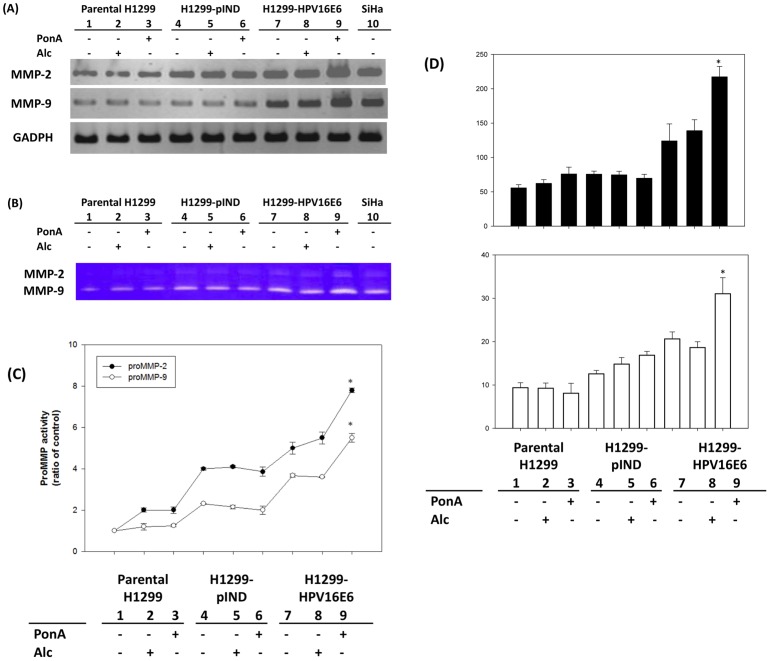
HPV16 E6 up-regulates MMP-2 and MMP-9 expressions and activities. Expressions and activities of MMP-2 and MMP-9 in H1299-HPV16E6 induced by PonA were significantly higher than that of parental H1299, H1299-pIND and H1299-HPV16E6 treated with Alc or PonA after 24 hours. (A) RNA was isolated and subjected to RT-PCR analysis using specific primers to MMP-2 and MMP-9. (B) MMP-2 and MMP-9 activities in the culture supernatants were determined by Zymogram. HPV 16 E6-containing SiHa cervical cancer cells were used as positive control (lane 10). The quantitated results for MMP activities in (B) were shown in (C). (D) Levels of MMP-2 and MMP-9 in cell culture mediate was respectively analyzed by MMP-2- and MMP-9- specific ELISA kit (*P<0.05 by *t*-test).

As shown in Figure 3A, both MMP-2 and MMP-9 RNA levels of H1299-HPV16E6 under PonA induction was significantly higher than other counterparts (Fig. 3A). We next examined the activities of MMP-2 and MMP-9 by zymography because activities of MMPs family proteins require processing and may not parallel to their protein expression levels. Zymographic results demonstrated that MMP-2 and MMP-9 activities were significantly increased accompanied with E6 expressions (Fig. 3, B and C). Both the increased ratios of MMP-2 and MMP-9 (at about 1.6 folds) were comparable with their protein expression levels. Results from ELISA analysis also revealed that both the secretory MMP-2 and MMP-9 levels were significantly elevated to about 1.6∼1.7 folds in PonA-induced H1299-HPV16E6 cells (MMP-2∶217.08±15.48 ng/mL; MMP-9∶31.05±3.70 ng/mL), compared with that under Alc treatment (MMP-2∶138.65±16.22 ng/mL; MMP-9∶18.62±1.35 ng/mL, Figure 3D and Table S2). The above results suggested that basal HPV 16 E6 expression up-regulates MMPs expression. Taken the results together, it suggestes that the RNA, proteins and activities of MMP-2 and MMP-9 are significantly up-regulated by HPV 16 E6 in H1299 cells.

### MMP-2 and MMP-9 Expressions are Up-regulated by HPV16-induced IL-8

**Figure 4:**
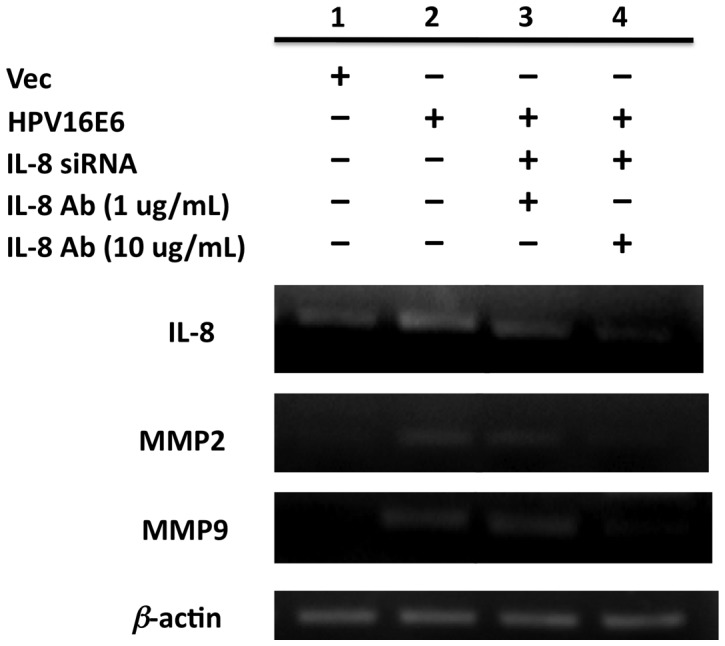
MMP-2 and MMP-9 expressions in H1299-HPV16E6 were decreased by inhibiting HPV-induced IL-8. H1299-HPV16E6 cells were cultured in RPMI 1640 with or without 5* u*M PonA and 100% Alc for 24 hours, followed by transfecting with IL-8 siRNA (lane 3 & 4) in the presence of 1 ug or 10 ug IL-8 antibodies for 6 hours to inhibit HPV-induced IL-8. MMP-2 and MMP-9 RNA was then examined by RT-PCR after 48 hours of further incubation.

To verify whether the HPV 16 E6-induced IL-8 was responsible for the increased MMP-2 and MMP-9 expressions, MMPs levels in HPV 16 E6-expressing cells were further examined under the combined treatment of IL-8 siRNA to antagonize its transcription and specific antibodies to neutralize the activities. As shown in Figure 4, both MMP-2 and MMP-9 expressions were decreased when HPV-induced IL-8 was inhibited. These results suggest that MMPs up-regulation in HPV16-infected lung cancer cells is at least in part mediated by IL-8.

## Discussion

The epidemiology of lung cancer remains partially unresolved since vast majority of smokers do not develop such tumors while at least 10–15% of lung cancers occur in non smokers [25]. Thus, factors other than smoking may also have an impact as etiological and risk factors for lung cancer. Our previous studies suggested that HPV may be involved in lung cancer development through up-regulating the expressions of IL-6, IL-17 and the anti-apoptotic Mcl-1, and result in promotion of cancer cell growth [8], [9]. In addition to inactivating p53 tumor suppressors and promoting the expressions of cell proliferation proteins, HPV infection is also associated with increased tissue angiogenesis mediated by inducing the expression of various pro-angiogenic molecules including IL-8 and MMPs in cervical cancer [15], [26]–[30]. Accordingly, the goal of the present study was to determine whether HPV infection in lung adenocarcinoma cells can promote the expression of angiogenesis- and metastasis- related proteins, including IL-8 and MMPs, to make the transformed cells equipped with angiogenic and metastatic characteristics.

Our results demonstrate that HPV E6 antigens alone significantly up-regulate IL-8 expression (Fig. 2 & Table S1). In addition, not only the expression of MMP-2 and MMP-9, but also their corresponding gelatinase activities are increased (Fig. 3 & Table S2). Our data further reveal that the increased MMP-2 and MMP-9 expressions are mediated by the HPV E6-induced IL-8 because the MMPs levels are reduced when HPV-induced IL-8 is inhibited by combined treatment of IL-8 siRNA and neutralizing antibodies (Fig. 4). These observations support previous studies in which IL-8 is up-regulated following expression of HPV 16 E6/E7 in primary foreskin keratinocytes and cervical cancer cell lines [15], [31]. Besides, our results also indicate that, in addition to cell proliferation-related machinery, HPV infection can promote the expression and activities of angiogenic and metastatic molecules in lung cancer cells. The combination of growth-promoting, angiogenic and metastatic machineries further confer the malignant and tumorigenic potentials on the infected cancer cells.

Inactive p53 and high-risk HPV infection are both associated with changes in angiogenesis by a p53-dependent pathway [26]. The increased angiogenesis and down-regulated apoptosis further facilitate tumor progression. On the contrary, HPV-regulated angiogenesis can alternatively occur through a p53-independent mechanism: the induction of VEGF transcription by HPV E6 remains equal in p53^−/−^ fibroblasts [32]. The present observation that HPV 16 E6 in p53^−/−^ IL-6-null H1299 cells can significantly up-regulate pro-angiogenic MMPs expressions through IL-8 and our previous reports in which E6 can induce IL-17 expressions [9] indicate that HPV 16 E6 can mediate angiogenic promotion and antiapoptotic effects through cytokines by a p53-independent mechanism.

IL-8 is a potent angiogenic factor and autocrine growth factor in several human cancers [11], which is associated with metastasis [12]–[14]. IL-8 promotes human lung cancer growth through its angiogenic properties [33]. Its autocrine and paracrine function has been shown to play an important role in angiogenesis, tumor growth, and metastasis [24], [25], [34], [35]. Recent reports suggest that IL-8 regulates the expression and activity of MMP-9 in human prostate carcinoma cell lines [24]. Moreover, IL-8 blocking antibody is able to down-regulate MMP-9 expression and activity in orthotopic bladder cancer xenografts [36]. IL-8 can also stimulate neutrophils to release stored MMP-9, which has been shown to exert positive feedback control by cleavage of IL-8, thus increasing its chemotactic activity for neutrophils [37], [38]. Expression of IL-8 by human melanoma cells up-regulates MMP-2 activity to increase tumor growth and metastasis [14]. IL-8 promotes the invasiveness of endometrial stromal cells to the extracellular matrix by upregulating MMP-2 and MMP-9 activity [39]. Furthermore, IL-8 is involved in the degradation of extracellular matrix by MMP-2 and MMP-9 activity, leading to endothelial cell migration, invasion and capillary tube organization [25]. Our results, in support of the above findings, suggest that IL-8 may act as an autocrine and/or paracrine angiogenic factor for HPV16 E6-expressing H1299 cells by enhancing MMP-2 and MMP-9 expression.

Although abundant evidences demonstrate that IL-8 expression is induced by HPV infection in several cancer cell lines, controversial results still exist. Huang et al. reported that HPV 16 E6 down-regulates IL-8 expression through NF-*k*B pathway in primary keratinocytes, which is contradictory to our observations [40]. Two possible factors may explain this discrepancy: First of all, the cell model in their study is primary cell while ours is lung adenocarcinoma. The nature or difference of these 2 cell models, and thus the regulatory machineries and gene compositions, may result in the conflicting results. Secondly, the cell-type specific gene expression patterns in response to a particular pathogen may lead to the differential results. Nevertheless, HPV 16 E6 has been reported to contribute to the induction of several pro-angiogenic factors, including IL-8, in human foreskin keratinocytes [30] and human umbilical vein endothelial cells [15]. Particularly, our observation supports a recent report from Li et al. [41], in which HPV 16 E6 overexpression in lung cancer cells significantly promotes angiogenesis via enhancing the expression of several pro-angiogenic factors such as HIF-1a, VEGF and IL-8.Earlier studies suggest that IL-8-mediated inhibition of apoptosis could be dependent or independent of Bcl-2 expression [25], [42]. IL-8 might regulate angiogenesis by modulating the anti-apoptosis pathway of CXCR1- and CXCR2- expressing endothelial cells [25]. Additionally, it is known that the anti-apoptotic Mcl-1 can also be up-regulated at both transcript and protein levels by IL-8 [43], and the loss of Mcl-1 expression in human polymorphonuclear leukocytes promotes apoptosis [44]. Some studies have demonstrated that IL-8 can regulate polymorphonuclear leukocytes apoptosis by up-regulating Mcl-1 antiapoptotic effect [42]–[45]. These data may suggest that IL-8 might also have anti-apoptotic activity, as the scenario of IL-6, by promoting Mcl-1 expression through its autocrine and/or paracrine functions by CXCR1 and/or CXCR2 receptor in HPV16 E6- expressing H1299 cells. However, this speculation awaits further study.

**Figure 5:**
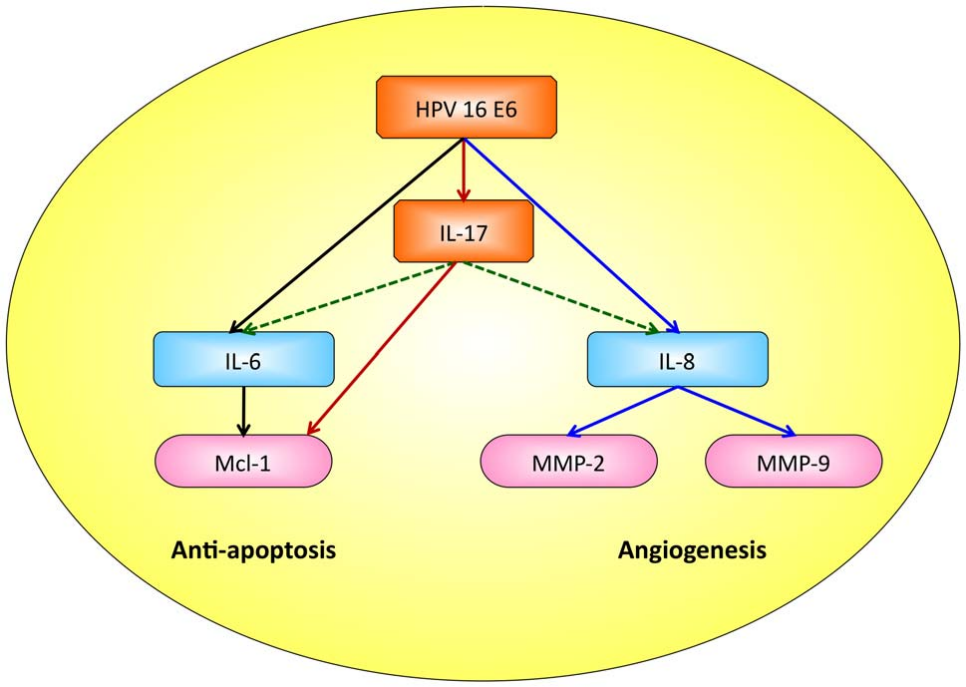
Elevated cytokines induced by HPV contribute to potentiate the proliferation and angiogenesis required for lung tumorigenesis. HPV-related lung tumorigenesis is suggested to be associated with the up-regulation of anti-apoptotic Mcl-1 expression mediating by IL-6. In addition, HPV up-regulates IL-17 which subsequently promotes Mcl-1 expression bypassing the IL-17 downstream IL-6 molecules. HPV-induced IL-8 may act as an angiogenic factor which augments MMPs production and activities.

In summary, our results reveal that the HPV 16 E6 significantly up-regulates pro-angiogenic MMP-2 and MMP-9 expressions through inducing IL-8 in a p53-independent manner. The present study suggests that HPV infection may be involved in angiogenic promotion mediated by IL-8, MMP-2, MMP-9 up-regulation in lung adenocarcinoma. Combining the present study and our previous data [8], [9], a model for high-risk HPV infection to potentiate the proliferating and angiogenic activities in lung cancer cells is proposed as depicted in Fig. 5. First of all, HPV infection induces IL-6 and Mcl-1 expression, which subsequently promotes lung tumorigenesis. Therefore, the HPV-related lung tumorigenesis is suggested to be associated with the up-regulation of anti-apoptotic Mcl-1 expression mediating by IL-6 (black pathway in Fig. 5) [8]. In addition, HPV 16 E6 oncoproteins induce IL-17 levels which subsequently promote the expression of anti-apoptotic protein Mcl-1 bypassing the IL-17-downstream IL-6 molecules through PI3K pathway in a p53-independent manner. The HPV-mediated IL-17 and Mcl-1-dependent anti-apoptotic effects may play an important role in HPV-associated lung tumorigenesis (red pathway in Fig. 5) [9]. Thirdly, HPV-induced IL-8 may act as an autocrine and/or paracrine angiogenic factor which augments MMPs production and activities (blue pathway in Fig. 5). In addition, we speculate that HPV infection may elicit a cytokine cascade by up-regulating IL-6 and IL-8 production through inducing IL-17, which is known to induce the release of IL-6 and IL-8 through the p38 and ERK MAPK pathway in human bronchial epithelial cells (green pathway in Fig. 5) [46], [47]. However, this speculation needs further investigation. Taken altogether, our studies provide the evidence of an association between inflammation and HPV infection in lung cancer. The cytokines induced by HPV may work together and contribute to potentiate the proliferation and angiogenesis required for lung tumorigenesis.

## Supporting Information

Table S1 **IL-8 levels were induced in HPV 16 E6-transfected H1299 cells.**
(DOC)

Table S2 **MMP-2 and MMP-9 levels were induced in HPV 16 E6-transfected cells.**
(DOC)
