# Correction: Human Papillomavirus Up-Regulates MMP-2 and MMP-9 Expression and Activity by Inducing Interleukin-8 in Lung Adenocarcinomas

**DOI:** 10.1371/annotation/a50fa759-93fb-444d-9523-9d4a805ef898

**Published:** 2013-06-07

**Authors:** Ming-Yuh Shiau, Li-Ching Fan, Shun-Chun Yang, Chang-Hui Tsao, Huei Lee, Ya-Wen Cheng, Li-Chuan Lai, Yih-Hsin Chang

There was an error in the affiliation of the first author. The correct affiliation is: Department of Nursing, College of Medicine & Nursing, Hungkuang University, Taichung, Taiwan, Republic of China 

