# Supplementary material for: Human Papillomavirus Up-Regulates MMP-2 and MMP-9 Expression and Activity by Inducing Interleukin-8 in Lung Adenocarcinomas
Source: PLoS One. 2013 Jan 21;8(1):e54423. doi: 10.1371/journal.pone.0054423 (PMC3549962; doi:10.1371/journal.pone.0054423)
Supplement: Table S1 — IL-8 levels were induced in HPV 16 E6-transfected H1299 cells. (DOC) [file pone.0054423.s001.doc]

**Table S1. IL-8 levels were induced in HPV 16 E6-transfected H1299 cells.**

| **Cell/Treatment** | **IL-8 (pg/mL)** | **Lane No. In Fig. 2B** |
| --- | --- | --- |
| Parental H1299 | 63.40 + 2.86 | 1 |
| Alc | 63.22 + 1.26 | 2 |
| PonA | 65.66 + 2.25 | 3 |
|  |  |  |
| H1299-pIND | 63.68 + 2.70 | 4 |
| Alc | 65.77 + 2.12 | 5 |
| PonA | 63.68 + 3.16 | 6 |
|  |  |  |
| H1299-HPV16E6 | 117.10 + 15.24 | 7 |
| Alc | 165.53 + 13.10 | 8 |
| PonA | 270.59 + 19.88 | 9 |
