# Supplementary material for: Human Papillomavirus Up-Regulates MMP-2 and MMP-9 Expression and Activity by Inducing Interleukin-8 in Lung Adenocarcinomas
Source: PLoS One. 2013 Jan 21;8(1):e54423. doi: 10.1371/journal.pone.0054423 (PMC3549962; doi:10.1371/journal.pone.0054423)
Supplement: Table S2 — MMP-2 and MMP-9 levels were induced in HPV 16 E6-transfected cells. (DOC) [file pone.0054423.s002.doc]

**Table S2. MMP-2 and MMP-9 levels were induced in HPV 16 E6-transfected cells.**

| **Cell/Treatment** | **MMP-2 (ng/mL)** | **MMP-9 (ng/mL)** |
| --- | --- | --- |
| Parental H1299 | 55.38 + 5.04 | 9.38 + 1.14 |
| Alc | 61.96 + 5.77 | 9.26 + 1.20 |
| PonA | 75.70 + 10.17 | 8.07 + 2.32 |
|  |  |  |
| H1299-pIND | 75.37 + 4.73 | 12.56 + 0.80 |
| Alc | 74.39 + 5.48 | 14.82 + 1.50 |
| PonA | 69.46 + 6.00 | 16.87 + 0.88 |
|  |  |  |
| H1299-HPV16E6 | 123.74 + 25.09 | 20.63 + 1.61 |
| Alc | 138.65 + 16.22 | 18.62 + 1.35 |
| PonA | 217.08 + 15.48 | 31.05 + 3.70 |
